# Supplementary material for: Modeling glioblastoma heterogeneity as a dynamic network of cell states
Source: Mol Syst Biol. 2021 Sep 16;17(9):e10105. doi: 10.15252/msb.202010105 (PMC8444284; doi:10.15252/msb.202010105)
Supplement: Supplementary file 5 — Source Data for Figure 3 [file MSB-17-e10105-s001.zip › Figure3A_sourcedata/GSEA_3065/hallmarks_state3.GseaPreranked.1623416274744/HALLMARK_HYPOXIA.html]

Details for gene set HALLMARK\_HYPOXIA[GSEA]

|  || Dataset | state3 |
| Phenotype | NoPhenotypeAvailable |
| Upregulated in class | na\_neg |
| GeneSet | HALLMARK\_HYPOXIA |
| Enrichment Score (ES) | -0.24946769 |
| Normalized Enrichment Score (NES) | -0.9210887 |
| Nominal p-value | 0.62666667 |
| FDR q-value | 0.75550973 |
| FWER p-Value | 1.0 |
Table: GSEA Results Summary

  

Fig 1: Enrichment plot: HALLMARK\_HYPOXIA      
 Profile of the Running ES Score & Positions of GeneSet Members on the Rank Ordered List

  

| PROBE | GENE SYMBOL | GENE\_TITLE | RANK IN GENE LIST | RANK METRIC SCORE | RUNNING ES | CORE ENRICHMENT || 1 | BCAN |  |  | 0 | 0.883 | 0.0655 | No |
| 2 | HSPA5 |  |  | 43 | 0.447 | 0.0938 | No |
| 3 | GPC1 |  |  | 90 | 0.355 | 0.1148 | No |
| 4 | COL5A1 |  |  | 302 | 0.242 | 0.1084 | No |
| 5 | PRKCA |  |  | 328 | 0.234 | 0.1229 | No |
| 6 | HS3ST1 |  |  | 377 | 0.223 | 0.1339 | No |
| 7 | WSB1 |  |  | 435 | 0.212 | 0.1430 | No |
| 8 | SDC3 |  |  | 539 | 0.194 | 0.1454 | No |
| 9 | SLC2A1 |  |  | 587 | 0.188 | 0.1539 | No |
| 10 | AKAP12 |  |  | 640 | 0.180 | 0.1613 | No |
| 11 | P4HA1 |  |  | 649 | 0.179 | 0.1736 | No |
| 12 | MYH9 |  |  | 688 | 0.173 | 0.1820 | No |
| 13 | HEXA |  |  | 731 | 0.166 | 0.1895 | No |
| 14 | ILVBL |  |  | 911 | 0.148 | 0.1797 | No |
| 15 | SLC2A3 |  |  | 1026 | 0.136 | 0.1767 | No |
| 16 | TPST2 |  |  | 1028 | 0.136 | 0.1866 | No |
| 17 | TMEM45A |  |  | 1038 | 0.135 | 0.1956 | No |
| 18 | MAP3K1 |  |  | 1219 | 0.120 | 0.1837 | No |
| 19 | SLC37A4 |  |  | 1251 | 0.118 | 0.1888 | No |
| 20 | ZNF292 |  |  | 1262 | 0.117 | 0.1964 | No |
| 21 | GPC4 |  |  | 1512 | 0.102 | 0.1751 | No |
| 22 | KDELR3 |  |  | 1678 | 0.093 | 0.1629 | No |
| 23 | VEGFA |  |  | 1723 | 0.090 | 0.1645 | No |
| 24 | CA12 |  |  | 1739 | 0.089 | 0.1694 | No |
| 25 | SCARB1 |  |  | 1789 | 0.087 | 0.1702 | No |
| 26 | GAA |  |  | 1823 | 0.085 | 0.1727 | No |
| 27 | SDC2 |  |  | 1875 | 0.082 | 0.1729 | No |
| 28 | KDM3A |  |  | 1980 | 0.077 | 0.1665 | No |
| 29 | IGFBP3 |  |  | 2112 | 0.071 | 0.1567 | No |
| 30 | B3GALT6 |  |  | 2152 | 0.070 | 0.1573 | No |
| 31 | SDC4 |  |  | 2189 | 0.069 | 0.1583 | No |
| 32 | PGM2 |  |  | 2408 | 0.060 | 0.1375 | No |
| 33 | EGFR |  |  | 2416 | 0.060 | 0.1412 | No |
| 34 | KLHL24 |  |  | 2486 | 0.057 | 0.1374 | No |
| 35 | CXCR4 |  |  | 2543 | 0.055 | 0.1350 | No |
| 36 | VLDLR |  |  | 2734 | 0.049 | 0.1167 | No |
| 37 | TGFBI |  |  | 2753 | 0.049 | 0.1182 | No |
| 38 | RBPJ |  |  | 2897 | 0.044 | 0.1049 | No |
| 39 | ERO1A |  |  | 3035 | 0.040 | 0.0920 | No |
| 40 | SERPINE1 |  |  | 3090 | 0.039 | 0.0886 | No |
| 41 | SRPX |  |  | 3518 | 0.028 | 0.0412 | No |
| 42 | STC2 |  |  | 3529 | 0.027 | 0.0421 | No |
| 43 | SIAH2 |  |  | 3635 | 0.025 | 0.0318 | No |
| 44 | PAM |  |  | 3683 | 0.023 | 0.0280 | No |
| 45 | SLC6A6 |  |  | 3732 | 0.022 | 0.0241 | No |
| 46 | NDST1 |  |  | 3755 | 0.021 | 0.0231 | No |
| 47 | ANKZF1 |  |  | 3816 | 0.020 | 0.0176 | No |
| 48 | FOS |  |  | 3899 | 0.018 | 0.0094 | No |
| 49 | UGP2 |  |  | 3907 | 0.017 | 0.0099 | No |
| 50 | CHST3 |  |  | 3949 | 0.016 | 0.0064 | No |
| 51 | CCNG2 |  |  | 4001 | 0.015 | 0.0016 | No |
| 52 | PFKP |  |  | 4040 | 0.014 | -0.0018 | No |
| 53 | PLAUR |  |  | 4075 | 0.013 | -0.0047 | No |
| 54 | PGM1 |  |  | 4098 | 0.013 | -0.0063 | No |
| 55 | JMJD6 |  |  | 4105 | 0.012 | -0.0061 | No |
| 56 | DUSP1 |  |  | 4186 | 0.010 | -0.0146 | No |
| 57 | DTNA |  |  | 4203 | 0.009 | -0.0158 | No |
| 58 | DPYSL4 |  |  | 4376 | 0.005 | -0.0354 | No |
| 59 | VHL |  |  | 4504 | 0.001 | -0.0500 | No |
| 60 | P4HA2 |  |  | 4511 | 0.001 | -0.0506 | No |
| 61 | GBE1 |  |  | 4604 | -0.001 | -0.0612 | No |
| 62 | ENO2 |  |  | 4676 | -0.003 | -0.0692 | No |
| 63 | CDKN1C |  |  | 4860 | -0.007 | -0.0898 | No |
| 64 | FOXO3 |  |  | 4960 | -0.010 | -0.1006 | No |
| 65 | PPP1R15A |  |  | 4983 | -0.010 | -0.1024 | No |
| 66 | TPBG |  |  | 5003 | -0.011 | -0.1037 | No |
| 67 | DDIT4 |  |  | 5025 | -0.012 | -0.1053 | No |
| 68 | ADORA2B |  |  | 5176 | -0.015 | -0.1216 | No |
| 69 | BHLHE40 |  |  | 5331 | -0.019 | -0.1380 | No |
| 70 | HDLBP |  |  | 5601 | -0.027 | -0.1671 | No |
| 71 | KLF6 |  |  | 5627 | -0.028 | -0.1679 | No |
| 72 | NAGK |  |  | 5652 | -0.028 | -0.1686 | No |
| 73 | MXI1 |  |  | 5656 | -0.029 | -0.1668 | No |
| 74 | GPI |  |  | 5670 | -0.029 | -0.1662 | No |
| 75 | NR3C1 |  |  | 5724 | -0.030 | -0.1701 | No |
| 76 | CDKN1B |  |  | 5783 | -0.032 | -0.1744 | No |
| 77 | XPNPEP1 |  |  | 5786 | -0.032 | -0.1722 | No |
| 78 | PLIN2 |  |  | 5856 | -0.034 | -0.1777 | No |
| 79 | STC1 |  |  | 5905 | -0.036 | -0.1806 | No |
| 80 | HK1 |  |  | 6031 | -0.041 | -0.1920 | No |
| 81 | NDRG1 |  |  | 6040 | -0.041 | -0.1899 | No |
| 82 | ATF3 |  |  | 6159 | -0.045 | -0.2003 | No |
| 83 | ISG20 |  |  | 6399 | -0.053 | -0.2241 | No |
| 84 | ALDOA |  |  | 6448 | -0.054 | -0.2256 | No |
| 85 | FAM162A |  |  | 6572 | -0.060 | -0.2354 | No |
| 86 | ERRFI1 |  |  | 6678 | -0.065 | -0.2428 | No |
| 87 | GALK1 |  |  | 6708 | -0.066 | -0.2412 | No |
| 88 | CASP6 |  |  | 6728 | -0.067 | -0.2384 | No |
| 89 | PFKL |  |  | 6771 | -0.070 | -0.2381 | No |
| 90 | CAV1 |  |  | 6859 | -0.073 | -0.2427 | No |
| 91 | DDIT3 |  |  | 6918 | -0.077 | -0.2438 | Yes |
| 92 | SLC25A1 |  |  | 6932 | -0.077 | -0.2395 | Yes |
| 93 | IDS |  |  | 6974 | -0.079 | -0.2384 | Yes |
| 94 | FOSL2 |  |  | 6992 | -0.080 | -0.2344 | Yes |
| 95 | NEDD4L |  |  | 7025 | -0.083 | -0.2320 | Yes |
| 96 | TIPARP |  |  | 7051 | -0.084 | -0.2287 | Yes |
| 97 | RRAGD |  |  | 7087 | -0.086 | -0.2263 | Yes |
| 98 | PGK1 |  |  | 7115 | -0.088 | -0.2229 | Yes |
| 99 | HOXB9 |  |  | 7142 | -0.090 | -0.2193 | Yes |
| 100 | CHST2 |  |  | 7170 | -0.092 | -0.2156 | Yes |
| 101 | ENO1 |  |  | 7257 | -0.097 | -0.2183 | Yes |
| 102 | GLRX |  |  | 7283 | -0.099 | -0.2139 | Yes |
| 103 | IRS2 |  |  | 7285 | -0.099 | -0.2066 | Yes |
| 104 | CITED2 |  |  | 7430 | -0.111 | -0.2151 | Yes |
| 105 | SAP30 |  |  | 7564 | -0.123 | -0.2214 | Yes |
| 106 | TPD52 |  |  | 7598 | -0.128 | -0.2157 | Yes |
| 107 | JUN |  |  | 7746 | -0.146 | -0.2220 | Yes |
| 108 | CAVIN3 |  |  | 7800 | -0.152 | -0.2168 | Yes |
| 109 | HMOX1 |  |  | 7843 | -0.157 | -0.2100 | Yes |
| 110 | BNIP3L |  |  | 8044 | -0.192 | -0.2189 | Yes |
| 111 | GRHPR |  |  | 8064 | -0.196 | -0.2066 | Yes |
| 112 | PNRC1 |  |  | 8133 | -0.212 | -0.1987 | Yes |
| 113 | BTG1 |  |  | 8162 | -0.218 | -0.1857 | Yes |
| 114 | ANXA2 |  |  | 8206 | -0.230 | -0.1736 | Yes |
| 115 | CAVIN1 |  |  | 8208 | -0.231 | -0.1566 | Yes |
| 116 | MIF |  |  | 8377 | -0.286 | -0.1549 | Yes |
| 117 | CSRP2 |  |  | 8589 | -0.393 | -0.1502 | Yes |
| 118 | PRDX5 |  |  | 8591 | -0.395 | -0.1209 | Yes |
| 119 | GAPDH |  |  | 8615 | -0.418 | -0.0926 | Yes |
| 120 | MT2A |  |  | 8650 | -0.456 | -0.0627 | Yes |
| 121 | CDKN1A |  |  | 8657 | -0.461 | -0.0292 | Yes |
| 122 | TPI1 |  |  | 8709 | -0.538 | 0.0049 | Yes |
Table: GSEA details [plain text format]

  

Fig 2: HALLMARK\_HYPOXIA: Random ES distribution      
 Gene set null distribution of ES for **HALLMARK\_HYPOXIA**
